# A Langmuir-Blodgett Study of the Interaction between Amphotericin B and Lipids of *Histoplasma capsulatum*

**DOI:** 10.3390/membranes12050483

**Published:** 2022-04-29

**Authors:** Pedronel Araque-Marín, Andrea Naranjo Díaz, Luisa Fernanda Gómez Londoño, María del Pilar Jiménez Alzate, Francesco Castelli, Maria Grazia Sarpietro, Cristiano Giordani, Carlos Alberto Peláez Jaramillo

**Affiliations:** 1 School of Life Sciences, Universidad Escuela de Ingeniería de Antioquia (EIA), Envigado 055428, Colombia; pedronel.araque@eia.edu.co; 2 Grupo Interdisciplinario de Estudios Moleculares, Institute of Chemistry, Faculty of Exact and Natural Sciences, Universidad de Antioquia, Medellín 050010, Colombia; andrea.naranjo@udea.edu.co (A.N.D.); carlos.pelaez@udea.edu.co (C.A.P.J.); 3 Grupo Micología Médica, Department of Microbiology and Parasitology, Faculty of Medicine, Universidad de Antioquia, Calle 70 No. 52-21, Medellín 050010, Colombia; luisa.gomezl@udea.edu.co (L.F.G.L.); delpilar.jimenez@udea.edu.co (M.d.P.J.A.); 4 Department of Drug and Health Sciences, Università degli Studi di Catania, Viale A. Doria 6, 95125 Catania, Italy; fcastelli@unict.it; 5 Institute of Physics, Faculty of Exact and Natural Sciences, Universidad de Antioquia, Calle 70 No. 52-21, Medellín 050010, Colombia; cristiano.giordani@udea.edu.co; 6 Grupo Productos Naturales Marinos, Faculty of Pharmaceutical and Food Sciences, Universidad de Antioquia, Calle 70 No. 52-21, Medellín 050010, Colombia

**Keywords:** histoplasmosis, histoplasma capsulatum, lipid extracts, clinical isolate, environmental isolate, mycelial phase, amphotericin B, langmuir monolayers, molecular organization

## Abstract

*Histoplasma capsulatum* is a dimorphic, thermal, and nutritional fungus. In the environment and at an average temperature of 28 °C, it develops as a mold that is composed of infecting particles. Once in the host or in cultures at 37 °C, it undergoes a transition into the parasitic form. In the present work, we performed chemical extraction and characterization using chromatography techniques of the associated lipid composition of the external surface of the cell wall of the mycelial phase of two isolates of the *H. capsulatum*: one clinical and one environmental. Several differences were evidenced in the fatty acids in the phospholipid composition. Surface pressure–area isotherms and compression module curves of the Amphotericin B and lipid extract monolayers, as well as (AmB)-lipid extract mixed monolayers were recorded. Results show a high affinity of AmB towards lipid extracts. The most stable monolayers were formed by AmB + environmental with a mass ratio of 1:3 and AmB + clinical with a mass ratio of 1:2. Knowledge of the AmB aggregation processes at a molecular level and the characterization of the lipid extracts allows the possibility to understand the interaction between the AmB and the lipid fractions of *H. capsulatum*.

## 1. Introduction

Histoplasmosis is an endemic systemic mycosis of primary pulmonary involvement, though it often spreads to other organs, such as the reticulate endothelial system, among others [1]. The disease is acquired by inhaling microconidia and mycelial fragments, which constitute the infecting particles of the dimorphic fungus *Histoplasma capsulatum* (*H. capsulatum*) [2,3], whose natural habitat is the soil, especially those with the presence of bird and bat droppings [1,4]. At room temperature, the fungus develops as a mycelial constituted by hyaline septate hyphae, which reproduces asexually, producing tuberculate macroconidia and microconidia.

Histoplasmosis is endemic in most of the world. However, it is on the American continent that it has greatest incidence, especially among agriculturists, farmers, builders, explorers, and speleologists, for whom the possibilities of contact with the pathogen are greater [1]. Previously, mycosis was considered rural, but current reports show outbreaks in urban areas, due to new constructions in forest areas, clearing of trees, demolitions, and the use of organic fertilizers, such as non-stabilized poultry manure [5,6,7].

The infection with *H. capsulatum* is considered an accidental event that occurs when a contaminated source is disturbed and the infecting particles are dispersed, and once inhaled, reach the alveolus, where they undergo the transition into parasitic form [2,8]. The development of histoplasmosis depends on the conditions of both the host and the fungus. In the case of the host, it will depend on the status of the immune system and the lung’s structural defects, and in the case of the fungus, it will depend on the amount of *H. capsulatum* inhaled inoculum and its virulence [2,9].

Studies have been conducted regarding the lipid contents of the fungus to better understand the virulence of *H. capsulatum* [10], which have stated a possible correlation between the lipid content and the fungus virulence. Until now, there have been some alternatives regarding the identification, isolation, and characterization of the lipid composition of the mycelial and yeast phases [11] and the extracellularly secreted vesicles [12] to establish the relation with the pathogenicity [13,14,15,16].

Amphotericin B is the initial therapy to treat the moderately severe to severe clinical forms of histoplasmosis for its fungicidal effect [17,18,19]. The action mechanism of amphotericin B is based on its union to the ergosterol of the cellular membrane of the fungus, generating the formation of channels that facilitate the outflow of the cytoplasmic content and consequential fungal cell death [20,21]. However, its adverse effects and toxicity occasionally require the interruption of the treatment with amphotericin B despite the presence of a severe fungal infection. Its principal chronic toxicity manifests as kidney failure, hypokalemia, hypomagnesemia, metabolic acidosis, and secondary polyuria to diabetes insipidus [22].

In the last decade, substantial progress has been made in the understanding of the organization and biological activity of amphotericin B in presence of sterols in lipid environments, centered in the interactions of amphotericin B with lipids and sterols, the formation of amphotericin B channels in membranes, and aggregation of amphotericin B molecules [21]. Many studies to improve the amphotericin B toxicity profile have focused on the preparation of liposomal formulations for the treatment of histoplasmosis [23,24]. The study of amphotericin B liposomes refers to the study of monolayers in the water–gas interface, monolayers deposited in the solid substrate by means of the Langmuir–Blodgett technique, micelles, vesicles, and multilayers [21].

This work structurally compares the lipid composition associated with the external surface of the cell wall in the mycelial phase of environmental and clinical isolates of *H. capsulatum*. In addition, the variation in molecular packaging between the lipid extracts associated with the external surface of the cell wall in the mycelial phase of *H. capsulatum* of the environmental and clinical isolates and amphotericin B, used in the treatment of severe clinical forms of histoplasmosis, has been studied.

It is expected that the results of this work allow to comprehend the possible interaction relation between antifungal molecules such as the amphotericin and the cell wall, not only of *H. capsulatum*, but also of other fungal pathogens.

## 2. Materials and Methods

### 2.1. Reagents

All solvents used in this study were high performance liquid chromatography (HPLC) appropriate for Sigma-Aldrich mass spectrometry (St. Louis, MO, USA). Amphotericin B (AmB) European Pharmacopoeia (EP) Reference Standard was purchased from Sigma-Aldrich (St. Louis, MO, USA).

### 2.2. H. capsulatum Culture

The clinical isolate used was *H. capsulatum* 17183, (equivalent to COL_H_024*), belonging to the fungal collection of the Grupo Micología Médica of the Department of Microbiology and Parasitology of the Faculty of Medicine of Universidad de Antioquia. This isolate was originally obtained from a patient’s biopsy, analyzed, and identified by the Grupo Micología Médica. The environmental isolate used was *H. capsulatum* 316-1 (equivalent to COL_S_1*), recovered and identified by the Grupo Micología Médica from a raw material sample (poultry manure) for the production of soil improvers, collected for the Grupo Interdisciplinario de Estudios Moleculares (GIEM) from the Institute of Chemistry of the Faculty of Exact and Natural Sciences of the Universidad de Antioquia. The isolates were kept in their mycelial phase with monthly subcultures in a Mycosel-MyC medium (Mycosel Agar, BBLTM; [ref. 211462]. Franklin Lakes, NJ, USA) and incubated at 23 °C.

### 2.3. Deactivation of H. capsulatum

Given that a *H. capsulatum* infection can occur after the inhalation of infecting particles that, once inhaled, reach the alveolus, where they are converted into parasitic form [2,8], it is important to make the fungus inactive before manipulation in the lipid extraction process. In order to make inactive the clinical and environmental isolates, a hexane solvent was used as a non-destructive inhibitor, which has already been employed in the inhibition of the dimorphic fungal pathogen *Coccidioides* spp. [25].

### 2.4. Extraction of Lipids

The lipids were extracted from the external surface of the cell wall of the mycelial phase of the environmental and clinical isolates. The mycelial fragments were mixed with hexane solvent at 23 °C. The *H. capsulatum* growth was controlled monthly in a culture medium to verify fungal viability, and then the inactive mycelial fragments were subject to Soxhlet extraction, using hexane as the primary solvent. A second extraction was carried out with chloroform, followed by a third extraction with isopropanol, and lastly with methanol. Reflux time was 2 h. Then, the extracts were concentrated by rotary evaporation, then further dried under a nitrogen gas stream and stored at −20 °C [26,27,28].

### 2.5. Preparation of Fatty Acid Methyl Esters (FAMEs) (Fatty Acid Methylation)

The different lipid fractions were treated with 4 mL of sodium hydroxide (NaOH) 0.5 M in methanol and water at 90 °C under a reflux system for a total of 7 min. Subsequently, 5 mL of boron trifluoride (BF_3_) were added at 20% (*v*/*v*) in methanol and were kept under a reflux system for a total of 2 min. The samples were mixed with 4 mL of heptane and kept under a reflux system for an additional minute. Samples were withdrawn from the water and let to set for 5 min. Finally, 100 mL of a sodium chloride saturated solution (NaCl) were added to improve the separation of the organic phase. Afterwards, the organic phases were deposited into Eppendorf tubes with anhydrous sodium sulfate to remove any water traces. The Eppendorf tubes were then centrifuged at 13,000 rpm for 7 min, and 200 µL of the supernatant sample was collected in automatic sampling glass lid vials (Agilent, Santa Clara, CA, USA) to be subjected to gas chromatography (GIEM Group Internal Protocol) [29].

### 2.6. Analysis of Fatty Acid by Gas Chromatography (GC Analysis of Fatty Acids)

The mixture of internal standards of fatty acid methyl esters in n-heptane (caproate, laurate, myristate, palmitate, stearate, oleate, linoleate, linolenate, arachidonate) was run before each test to estimate retention times, followed by the different extracts, which were carried out independently, using Agilent 6890 N gas chromatography with a flame ioniza-tion detector (FID, Santa Clara, CA, USA, EE. UU.). A Split/Splitless Injection System and Agilent 7683 Auto-injector, with 50 m DB 23 capillary column, 0.25 internal diameter, and a 1.4 µm thick film was used. The chromatography flow was carried out on a FAME column (Model FAME JIWDB23, Agilent) with hydrogen gas. The method used was the GC-AOAC Official methods 996.06 and the data were analyzed using the Agilent ChemStation software using the areas under the curve for each analyte. The unsaturated/saturated relation was determined by means of the relation of the sum of the unsaturated fatty acids and the sum of the saturated fatty acids in each isolate [30].

### 2.7. Analysis of the Phospholipids and Glycolipids by Ultra High-Performance Liquid Chromatography (UHPLC)

The lipid fractions were characterized by reverse phase liquid chromatography-mass spectroscopy/mass spectroscopy (LC-MS/MS), with an ACQUITY UPLC^®^ BEH C18 (2.1 × 100 mm, 1.7 μm) analytical column, and an acidified aqueous solution with 0.1% of formic acid (A) and acetonitrile (B); initiating with 100% (A), 85% (A) and 15% (B) from 0 to 0.25 min, until reaching 100% (B) in 7.75 min (remaining until 8.50 min); alternating until 85% and 15% (B) in 8.51 min (remaining until 16 min), with a 0.300 mL/min flow at a 5 μL injection rate, having an ESI +/− ionization source and as a detector the mass spectrometer, operating on MS/MS Mode. The spectra were collected in the range of 100–1500 *m*/*z* and were manually analyzed for the identification of lipid species.

### 2.8. Monolayer Studies

The Langmuir technique measures the surface pressure (π) as a function of the mean molecular area (A) that occupies a molecule in a monolayer extended on an aqueous surface [31,32]. Experimental measurements were recorded with a Nima Langmuir balance equipped with a Wilhelmy platinum plate (Nima Technology, Coventry, England) and a Teflon trough that was rinsed with ethanol and distilled water before use. All experiments were performed at 37 °C. Before each measurement, the sub-phase was checked to be clean and that the surface tension value did not exceed ±0.1 mN/m. The monolayer stability was verified by monitoring the change in surface pressure while the area was held constant.

### 2.9. Monolayer Compression Isotherms

The surface pressure-area (π–A) isotherms were recorded with a KSV minitrough apparatus (KSV instrument Ltd., Helsinki, Finland) provided with a computer interface unit, an operating software, a trough (24,225 mm^2^ available area) made of Teflon, and two mobile barriers made of delrin. Monolayers were formed by applying small drops of the spreading solutions with a microsyringe (Hamilton Co., Reno, NV, USA) on a buffer solution (TRIS 5 mM, pH 7.4) prepared with ultrapure water with a resistivity of 18.2 MΩ cm^−1^, obtained through a Simplicity 185 Millipore system. After 10 min (to allow the solvent evaporation), monolayers of the desired composition were continuously compressed at a rate of 1 cm/minute. The films were compressed to their collapse pressure. Each run was repeated three times and the reproducibility was ±1 Å^2^ molecule^−1^. To form the monolayers of the single components, solutions of AmB (0.447 g/L) were prepared in chloroform/methanol 1:1 (in volume), environmental lipids extracts (13.737 g/L) in chloroform, and clinical lipids extracts (9.277 g/L) in chloroform. Isotherms of AmB, environmental, and clinical lipids extracts were performed in increasing volumes from 10 μL to 80 μL, from 3 μL to 80 μL, and from 3 μL to 50 μL, respectively. In order to study the effect of the adsorption of AmB on the environmental and clinical lipids extracts and to establish a possible selective interaction with some of the lipid components, mixtures of AmB/environmental lipids extract and AmB/clinical lipids extract were prepared by keeping the volume of extracts constant (5 µL) and varying the AmB volume (20, 30, 40, 50, 60, 70, and 80 µL). Another set of experiments was carried out with monolayers in which the volume of AmB was left constant (50 µL) but the volume of the clinical or environmental lipids extract was varied (5, 10, 15, and 20 µL for both extracts).

### 2.10. Geometry Optimization

The structural model for the molecular organization of AmB in monolayer lipids was developed using a Spartan 18′ software (Wavefunction) purchased license (Wavefunction, 1991). The molecular optimization was conducted following minimum geometric and global energy parameters using the semi-empirical method Austin Model 1 (AM1). Wavefunction (1991). Spartan 18′. Wavefunction, Available at: https://www.wavefun.com/corporate/more_spartan.html (accessed on 1 October 2020).

### 2.11. Statistical Model of the Data Analysis

All experiments were performed with three independent replicates (*n* = 3) and the data were expressed as means ± standard deviation. The results were analyzed via one-way ANOVA, and significant differences between the mean values were determined using Duncan’s multiple range test (*p* < 0.05). The STATGRAPHICS Centurion XV statistical package (Statgraphics Technologies, Plains, VA, USA) was applied.

## 3. Results

The inactivation of both *H. capsulatum* isolates in mycelial phase was achieved after remaining in hexane solvent for five months, with sterility tests at 90 and 120 days.

### 3.1. Composition Analysis of Fatty Acids Associated to the External Surface of the Cell Wall in the Mycelial Phase of H. capsulatum by CG

**Figure 1:**
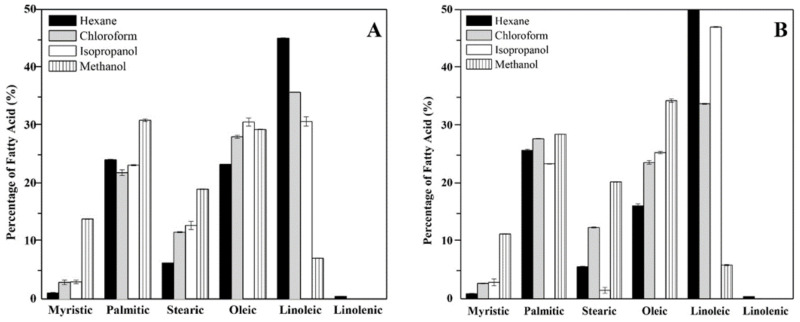
Fatty acid profile of the lipid extracts associated to the external surface of the cell wall in the mycelial phase of the environmental (**A**) and clinical (**B**) isolate of *H. capsulatum*.

The fatty acids associated to the external surface of the cell wall in the mycelial phase of the *H. capsulatum* environmental and clinical isolates were methylated and analyzed by GC. The fatty acids identified by comparison with a mix of external standards are the following: myristic acid (C14:0), palmitic acid (C16:0), stearic acid (C18:0), oleic acid (C18:1), linoleic acid (C18:2), and linolenic acid (C18:3). The fatty acid content in the environmental isolate was: myristic acid (5%), palmitic acid (25%), stearic acid (13%), oleic acid (28%), linoleic acid (27%), and linolenic acid (0.03%) (Figure 1A); while the fatty acid content found in the clinical isolate was: myristic acid (5%), palmitic acid (25%), stearic acid (8%), oleic acid (27%), linoleic acid (40%), and linolenic acid (0.02%) (Figure 1B).

Although there is a similar trend in the total fatty acid content in the environmental and clinical isolates, there is a significant statistical difference between the isolates (*p*-value < 0.05), particularly among the palmitic, stearic, oleic, and linolenic acids. Regarding the contribution of each solvent, the hexane and methanol fractions did not have any significant statistical difference among the isolates when comparing the unsaturated/saturated ratios (*p*-value < 0.05), while the fraction of isopropanol from the clinical isolate had the largest contribution in the unsaturated/saturated ratio. The unsaturated/saturated ratio of the clinical isolate is 20% greater than that of the environmental isolate.

### 3.2. Analysis of the Phospholipids Associated with the External Surface of the Cell Wall of the Mycelial Phase of H. capsulatum by ESI-MS

The phospholipids associated to the external surface of the cell wall in the mycelial phase of the environmental and clinical *H. capsulatum* isolates were ionized with a positive charge and identified by ionic fragment search. With a positive charge, the following acid species: phosphatidic (PA), phosphatidylethanolamine (PE), phosphatidylglycerol (PG), and phosphatidylinositol (PI), were identified as Na^+^ or Li^+^ adducts, or individually protoned. The detected phospholipids showed *m*/*z* ions between 290 *m*/*z* and 820 *m*/*z*. The PE, PC, and PA phospholipids were identified in the mycelial phase of the clinical and environmental isolates. A total of eleven and ten phospholipids were identified in the clinical and the environmental isolates, respectively. These results are shown in Table 1.

### 3.3. Surface Pressure Isotherms of AmB Monolayers

The solution that was prepared in order to form AmB monolayers in the Langmuir Teflon trough was: AmB (0.447 mg/mL) in chloroform/methanol 1:1 (V:V). AmB isotherms were then recorded at increasing volumes from 10 μL to 80 μL (Figure S1). The isotherm to represent the formation of monolayers of the AmB was determined at 50 μL [33,34,35].

### 3.4. Compression Isotherms of Monolayers of AmB

**Figure 2:**
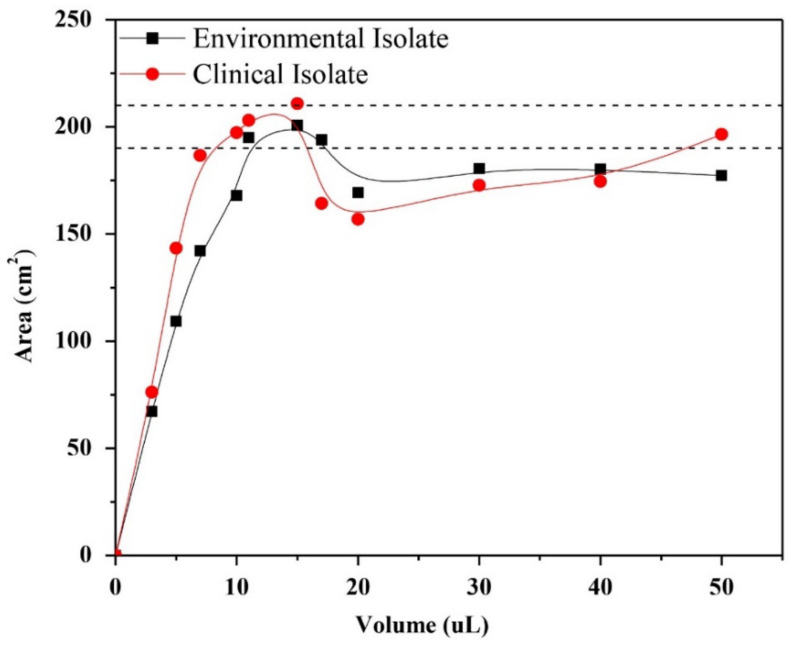
Effect of the volume of the lipid extracts associated to the external surface of the cell wall of the mycelial phase of *H. capsulatum* of the environmental and clinical isolates on the Teflon trough of the monolayer formed at a surface pressure of 5 mN/m.

The lipid extracts associated to the external surface of the cell wall of the mycelial phase of *H. capsulatum* of the environmental and clinical isolates were prepared in chloroform in concentrations of 13.737 g/L and 9.277 g/L, respectively. The isotherms were then made from the lipid extracts, in incremental volumes from 3 μL to 50 μL. Given the lack of knowledge of the isotherm to represent the formation of monolayers of the lipid extract, a surface pressure of 5 mN/m was determined as a constant, and by drawing a base line at 5 mN/m, it was possible to evaluate the effect of the volume of the extract on the formation area of the monolayer. In Figure 2, it is shown that the lipid extracts in volumes between 5 and 20 μL reach the maximum monolayer area formed between 190 and 210 cm^2^.

**Figure 3:**
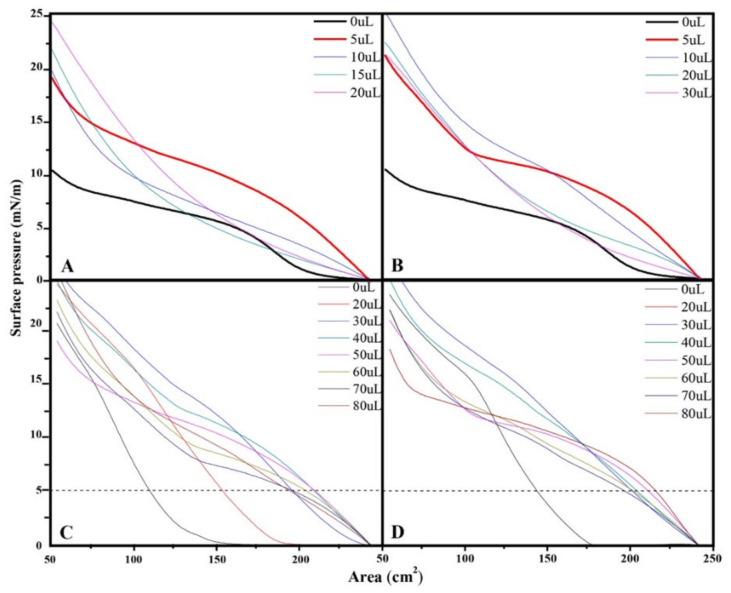
Surface pressure-area isotherms from the monolayer trough: (**A**); AmB (50 µL) with increasing volume of lipids extracts from environmental isolate; (**B**) AmB (50 µL) with increasing volume of lipid extract from clinical isolate; (**C**) environmental isolate extract (5 µL) with increasing volume of AmB; (**D**) clinical isolate extract (5 µL) with increasing volume of AmB. Concentrations are 13.737 g/L for environmental and 9.277 g/L for clinical extracts, respectively. The area values in Figure are given in cm^2^ (area occupied on the trough) because the molecular weight of the extracts is not known.

From the AmB isotherm at 50 µL, the effect of the volume of the lipid extracts associated to the external surface of the cell wall of the mycelial phase of *H. capsulatum* of the environmental and clinical isolates was evaluated, finding that the isotherm of 50 µL AmB + 0 µL of the environmental extract has a similar trend (parallel) to the isotherm of 50 µL AmB + 5 µL of the environmental extract (Figure 3A). Similarly, the isotherm of 50 µL AmB with 0 µL of the clinical extract has a similar trend to that of the isotherm of 50 µL AmB with 5 µL of the clinical extract (Figure 3B). The changes in surface pressure and the monolayer area indicate that there is a molecular reorganization in the subphase. Based on the relation of AmB (50 µL) with the lipid extracts (5 µL), the AmB effect was evaluated (20, 30, 40, 50, 60, 70, and 80 µL), maintaining the lipid extract constant (Figure 3C,D).

**Figure 4:**
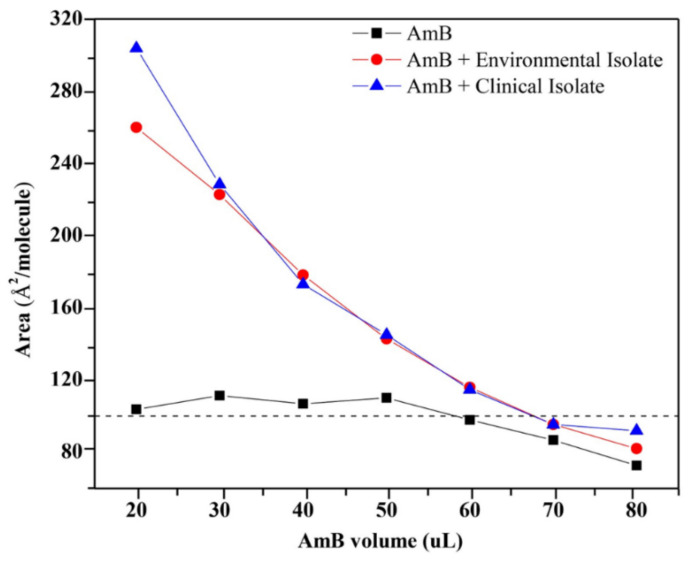
Effect of the volume of AmB on the area of the monolayers of lipids extracts associated to the external surface of the cell wall of the mycelial phase of *H. capsulatum* of the clinical (5 µL) and environmental (5 µL) isolates.

A surface pressure of 5 mN/m was determined as a constant, and by drawing a base line at 5 mN/m it was possible to evaluate the effect of the volume of amphotericin B on the area of the monolayer extracts at constant volume. In values greater than 50 µL, the AmB completely displaces the lipid molecules from the interface, presenting similar values to those of amphotericin in the absence of extracts (Figure 4).

**Figure 5:**
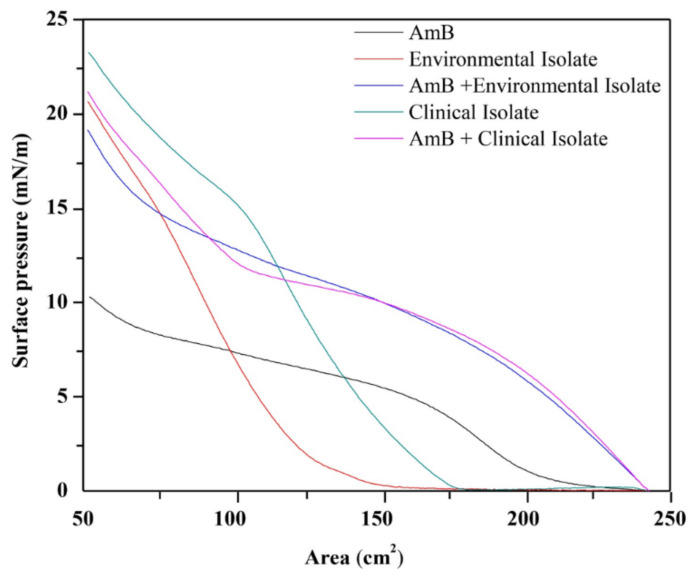
Surface pressure-area isotherms from the AmB monolayers trough (50 µL); lipids extracts associated to the external surface of the cell wall of the mycelial phase of *H. capsulatum* of the environmental (5 µL) and clinical (5 µL) isolates; mixture of AmB (50 µL) with lipid extract (5 µL). The area values in figure are given in cm^2^ (area occupied on the trough) because the molecular weight of the extracts is not known.

The surface pressure–area isotherms of the monolayers of AmB, of the lipid extracts associated to the external surface of the cell wall of the mycelial phase of *H. capsulatum*, and of the AmB/lipid extract mixed monolayers are shown in Figure 5. For the mixture of 50 µL of AmB with a concentration of 0.447 g/L with 5 µL of environmental lipid extract with a concentration of 13.737 g/L, there is a total volume of 55 µL with new concentrations of 0.406 g/L for AmB and 1.249 g/L for the environmental lipid extract, indicating an AmB mass/environmental lipid extract ratio of 1:3. For the mixture of 50 µL of AmB with a concentration of 0.447 g/L with 5 µL of clinical lipid extract with a concentration of 9.277 g/L, there is a total volume of 55 µL with new concentrations of 0.406 g/L for AmB and 0.843 g/L for the clinic lipid extract, indicating an AmB mass/clinical lipid extract ratio of 1:2.

## 4. Discussion

The clinical isolate obtained from the biopsy of a patient originally grew at a body temperature of 37 °C. The contact of *H. capsulatum* with the host produces an alteration in the fatty acid composition of both isolates. This alteration could be explained by the difference in epigenetic factors, firstly, remodeling the absorption of fatty acids; and secondly, controlling the enzyme activity involved in the synthesis of fatty acids [36]. The physiological and nutritional conditioning in the mycelial phase requires continuous regulation of the membrane lipids to achieve a constant flow under diverse growth conditions [30] due to the change in temperature (from 20 °C to 25 °C) and modification to the culture medium for mycelial development [10]. To this new condition of temperature and nutrients, the fatty acid biosynthesis is oriented to produce unsaturated fatty acids, allowing greater permeability and reducing the stiffness of the lipid membrane [37], thus conserving approximately the same flow rate.

Even though the lipids found are associated to the construction of cellular membranes (lipid bilayers), these lipids also carry out a diverse number of functions, from the compartmentalization of the cytoplasm to the storage of the protein involved in cell signaling, intercellular adhesion and cytoskeleton support [12]. Studies regarding the presence of phospholipids in *H. capsulatum* have been carried out mostly in parasitic form (yeast) [10,15,37,38,39,40,41,42] rather than its infecting form (microconidia and hyphal fragments: mycelial or saprophytic phase).

Amphotericin B is the main antifungal used to treat the moderately severe and severe clinical forms produced by *H. capsulatum*. Thus, it is useful to know the molecular interaction of amphotericin B and the external surface of the cell wall components of the target fungal cell.

**Figure 6:**
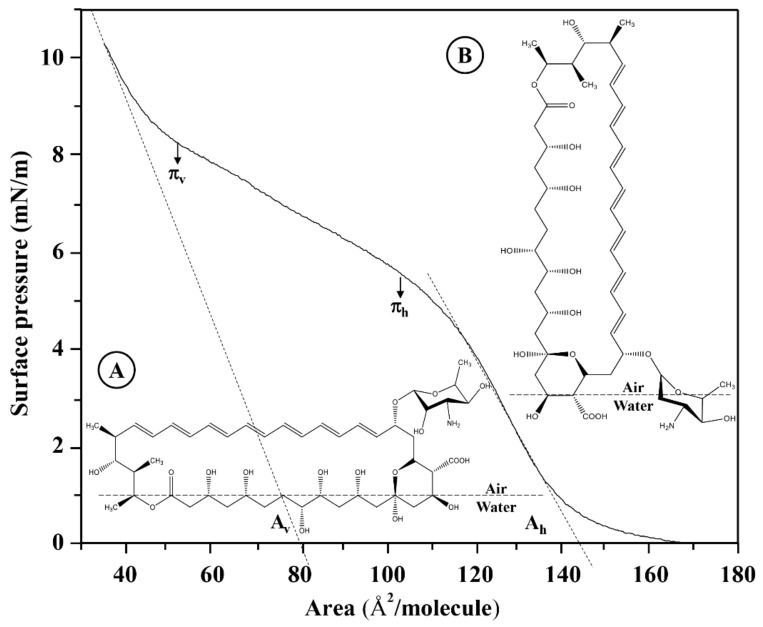
Surface pressure-area isotherms of AmB monolayers extended on Tris buffer 5 mM, pH = 7.4. The linear adjustments to the linear positions of the extrapolated compression isotherms to surface pressure zero point to the specific molecular areas in a horizontal position (A_h_) and in a vertical position (A_v_). The figure shows a subphase molecular reorientation model of AmB at a temperature of 37 °C. Concentration is 0.447 mg/mL. (**A**) horizontal position (Ah); (**B**) vertical position (Av).

The area per molecule of AmB on the Langmuir trough is calculated by the following Equation (1):

Å^2^/molecule = [Teflon trough area (Å^2^) · MM]/[Fc · NA · C · V] (1)

where the trough area range (50–250 cm^2^) in [Å^2^] is 5.0 × 10^17^–2.50 × 10^18^ Å^2^; NA is the number of Avogadro (6.022 × 10^23^ molecules/mol); MM is the molar mass of the AmB molecule (924.08 g/mol); C is the concentration of the AmB solution measured in [mg/mL]; V is the AmB solution volume measured in [µL], and Fc is the unit correction factor (1 × 10^−6^ mL·g/uL·mg). The isotherm at 50 μL and 0.447 g/L represents the reference system for the formation of the AmB monolayer in a range between 30 and 172 Å^2^, knowing that the linear adjustments to the linear positions of the extrapolated compression isotherms to surface pressure zero point to the specific molecular areas in a horizontal position (A_h_) and in a vertical position (A_v_) (Figure 6) [33,43,44].

The specific molecular areas of a horizontal position (A_h_) and vertical position (A_v_) do not depend only on the molecular reorientation model of the AmB molecules in the subphase. For the AmB molecule, a rectangular box type model has been proposed [21,33], as observed in Figure S2. Table 2 shows the experimental values of the specific molecular areas of AmB in horizontal (A_h_) and vertical (A_v_) positions compared to the values obtained by molecular optimization at AM1 theory level.

**Figure 7:**
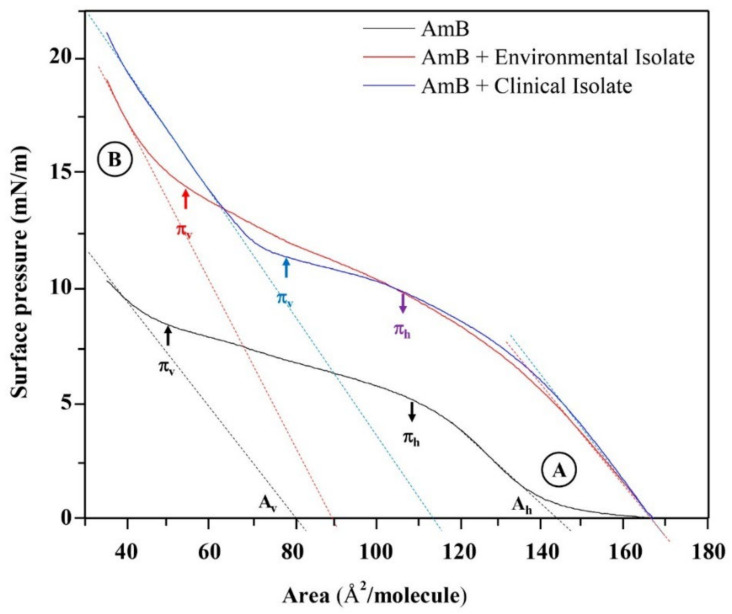
Surface pressure-area isotherms per molecule of Amphotericin B and the mixture of Amphotericin B with lipid extracts associated to the external surface of the cell wall of *H. capsulatum* mycelial phase extended on Tris buffer 5 mM, pH = 7.4. The linear adjustments to the linear positions of the extrapolated compression isotherms to surface pressure zero point to the specific molecular areas in a horizontal position (A_h_) and in a vertical position (A_v_). Concentrations are 13.737 g/L for environmental and 9.277 g/L for clinical extracts, respectively. (**A**) horizontal position (Ah); (**B**) vertical position (Av).

The AmB and AmB+ lipid extracted isotherms are represented in Figure 7. The linear adjustments to the linear positions of the extrapolated compression isotherms to surface pressure zero point to the specific molecular areas in a horizontal position (A_h_) and in a vertical position (A_v_), not only dependent on the reorientation model of the AmB molecules in the subphase, but also the packing of the AmB molecules and the lipid molecules that are found in each lipid extract. Table 2 shows the values corresponding to the molecular areas in horizontal (A_h_) and vertical (A_v_) positions with their corresponding surface pressures in horizontal (π_h_) and vertical (π_v_) positions.

**Figure 8:**
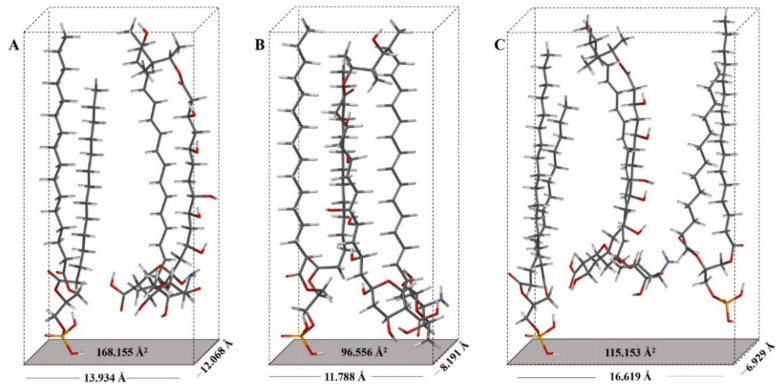
Orientation model of the AmB molecule with lipids extracts of the external surface of the cell wall of mycelial phase of *H. capsulatum*: (**A**) Environmental isolate in vertical position (A_v_); (**B**) Environmental isolate in horizontal position (A_h_). (**C**) Clinical isolate in horizontal position (A_h_).

When comparing the molecular areas and the surface pressures in the horizontal position (A_h_ and π_h_) of the AmB and AmB + lipid extract molecules, there was no significant difference between the evaluated lipids extracts. However, the differences between the molecular areas of the lipid extracts with AmB generated a value of 22 Å^2^/molecule. Using the phosphatidic acid (PA) as a model, being the forerunner of the phospholipids found in the lipid extracts of *H. capsulatum* and based on the rectangular box type model proposed for AmB [21,43], the area of the horizontal position (A_h_) of the phosphatidic acid molecule was calculated, resulting in a value of 20.382 Å^2^/molecule (Figure S3A,B). When comparing the molecular areas and surface pressures in the vertical position (A_v_ and π_v_) of the AmB and AmB + lipid extracted molecules, there were differences in area as well as in pressure. The difference between the molecular areas of the compared lipid extracts with AmB resulted in values of 10 Å^2^/molecule and 24 Å^2^/molecule for the environmental and clinical lipid extracts, respectively. The surface pressure of 14.2 mN/m for the AmB + environmental lipid extract mixture indicates a greater degree of packing. Figure 8 proposes the molecular packing model between AmB and the lipids of the external surface of the cell wall of the mycelial phase of the environmental and clinical *H. capsulatum* isolates. The calculation of the vertical position (A_v_) of the AmB molecule with phosphatidic acid produces a value of 96.556 Å^2^/molecule compared to the experimental value of 90 Å^2^/molecule.

The AmB + clinical lipid extract mixture produced a surface pressure of 11.4 mN/m, which is 2.8 mN/m less than that generated with the environmental lipid extract. The calculation of the area in the vertical position (Av) of the AmB molecule with phosphatidic acid produced a value of 115.153 Å^2^/molecule compared to the experimental value of 114 Å^2^/molecule. The calculation of the area in the horizontal position (A_h_) of the AmB molecule with phosphatidic acid produced a value of 168.155 Å^2^/molecule, and when compared to the experimental value of 166 Å^2^/molecule this indicates that the AmB molecule, when in the presence of lipid extracts, loses its horizontal position. The experimental values of the specific molecules of AmB and AmB + lipid extracts compared to the values obtained by molecular optimization to an AM1 level of theory with their respective energy minimization values are presented in Table 2.

## 5. Conclusions

Histoplasmosis is an endemic systemic mycosis in most of the world and the patients affected by this pathology are initially treated using amphotericin B (AmB), which is the drug usually employed to treat serious and potentially life-threatening fungal infections. It is of fundamental importance to study the interaction of both clinical and environmental fungi cell wall lipids extracts with AmB to identify the differences due to their different lipid compositions. We focused our study on the interaction of both extracts, using them to form lipid monolayers at the air–water interface in a Langmuir interfacial trough and allowing them to interact with AmB. This led us to determine that the surface coupling of AmB and the lipids extracted from the external surface of the cell wall of the mycelial phase of the environmental isolate was 1:3, while the relation between Am B and the lipids extracts associated to the external surface of the cell wall of the mycelial phase of the clinical isolate was 1:2. These results help to understand the possible interaction between anti-fungal molecules such as AmB and the cell wall, not only of *H. capsulatum*, but also of other fungal pathogens.

## Figures and Tables

**Table 1:** Composition of major phospholipids tentatively identified by ESI (positive-ion charge)-MS total ion associated to the external surface of the cell wall of the mycelial phase of the environmental and clinical isolates of *H. capsulatum* (ND: Not Detected).

Environmental Isolate		Clinical Isolate		Proposed Composition	Mass Prediction (Da)
Species	*m*/*z*	Species	*m*/*z*		
Phosphatidylethanolamine (PE)					
ND	ND	[M+Na]^+^	761.585	C18:3/C18:1	763.016
[M+H]^+^	663.455	[M+H]^+^	663.455	C14:0/C16:0	663.918
Phosphatidylcholine (PC)					
[M+Na]^+^	792.383	ND	ND	C16:0/C18:0	785.097
[M+H]^+^	737.546	ND	ND	C14:0/C18:0	734.057
[M+H]^+^	736.571	ND	ND	C18:3/C18:1	740.010
[M+H]^+^	708.511	[M+H]^+^	708.511	C14:0/C16:0	705.999
ND	ND	[M+H]^+^	701.493	C12:0/C18:2	701.967
ND	ND	[M+H]^+^	684.204	C10:0/C18:2	673.913
Phosphatidic Acid (PA)					
[M+Na]^+^	733.284	[M+Na]^+^	733.284	C18:1/C18:1	721.953
ND	ND	[M+Na]^+^	703.413	C18:0/C18:0	704.003
[M+H]^+^	684.203	[M+H]^+^	684.203	C18:2/C18:2	695.939
[M+Na]^+^	663.455	[M+Na]^+^	663.455	C14:0/C18:2	663.853
[M+Na]^+^	641.199	[M+Na]^+^	641.199	C14:0/C16:0	642.208
[M+Na]^+^	608.875	[M+Na]^+^	608.875	C10:0/C18:2	610.745

**Table 2:** Values of molecular areas in horizontal (A_h_) and vertical position (A_v_) of AmB and AmB + lipid extracts with their corresponding surface pressures in horizontal (π_h_) and vertical (π_v_) positions, compared to the values obtained by molecular optimization to an AM1 level of theory using SPARTAN Pro Software.

System	A_v_ (Å^2^/Molecule)	π_v_ (mN/m)	Energy (kJ/mol)	A_h_ (Å^2^/Molecule)	π_h_ (mN/m)	Energy (kJ/mol)
AmB (Experimental)	80	8.4	-	144	5.0	-
AmB (AM1 Calculated)	75.208	-	−3271.74	146.234	-	−3271.74
AmB + environmental lipid extract (Experimental)	90	14.2	-	166	9.8	-
AmB + environmental lipid extract (AM1 Calculated)	96.556	-	−5904.28	168.155	-	−5876.54
AmB + clinical lipid extract (Experimental)	114	11.4	-	166	9.8	-
AmB + clinical lipid extract (AM1 Calculated)	115.153	-	−8507.29	168.155	-	−5876.54

## Data Availability

Not applicable.

## Supplementary Materials

The following supporting information can be downloaded at: https://www.mdpi.com/article/10.3390/membranes12050483/s1, Figure S1: Surface pressure-area isotherms from the Teflon trough for the monolayers of AmB in incremental volume. Concentration is 0.447 g/L. The area values in Figure are given in cm^2^ (area occupied on the trough) because the molecular weight of the extracts is not known. Figure S2: Reorientation model of the AmB molecule: (A) horizontal position (Ah); (B) vertical position (Av). Energy Minimization to a value of −3271.74 kJ/mol, Figure S3: (A) Phosphatidic acid (PA) as the forerunner of the other phospholipids, (B) Phosphatidic acid in horizontal position (Ah) with its space-filling model and aerial views.

## References

[1] Jiménez R., Tobón A., Arango M., Espinal D., Restrepo A. (2002). Histoplasmosis: Utilidad de las pruebas serológicas en el diagnóstico diferencial. Acta Med. Colomb..

[2] Kauffman C. (2007). Histoplasmosis: A clinical and laboratory update. Clin. Microbiol. Rev..

[3] Abidon A.M. (2009). Chapter4—Histoplasmosis. J. Bras. Pneumol..

[4] Emmons C.W. (1954). Histoplasmosis: Animal reservoirs and other sources in nature of the pathogenic fungus, Histoplasma. Am. J. Public Health.

[5] Ordóñez Ν., Tobón A., Arango M., Tabares A., de Bedout C., Gómez B., Castanñeda E., Restrepo A. (1997). Brotes de histoplasmosis registrados en el área andina colombiana. Biomédica.

[6] Gómez L.F., Torres I.P., Jiménez M.D.P., McEwen J.G., de Bedout C., Peláez C.A., Acevedo J.M., Taylor M.L., Arango M. (2017). Detection of *Histoplasma capsulatum* in organic fertilizers by Hc100 nested PCR and its correlation with the physicochemical and microbiological characteristics of the samples. Am. J. Trop. Med. Hyg..

[7] Gómez L.F., Arango M., McEwen J.G., Gómez O.M., Rodríguez A.C., Peláez C.A., Acevedo J.M., Taylor M.L., Jiménez M.D.P. (2019). Molecular epidemiology of Colombian *Histoplasma capsulatum* isolates obtained from human and chicken manure samples. Heliyon.

[8] Porta A., Eletto A., Török Z., Franceschelli S., Glatz A., Vígh L., Maresca B. (2010). Changes in membrane fluid state and heat shock response cause attenuation of virulence. J. Bacteriol..

[9] Garfoot A.L., Rappleye C.A. (2015). *Histoplasma capsulatum* surmounts obstacles to intracellular pathogenesis. FEBS J..

[10] Nielsen H.S. (1966). Variation in lipid content of strains of *Histoplasma capsulatum* exhibiting different virulence properties for mice. J. Bacteriol..

[11] Zarnowski R., Dobrzyn A., NtAmBi J.M., Woods J.P. (2008). Neutral storage lipids of *Histoplasma capsulatum*: Effect of culture age. Curr. Microbiol..

[12] Albuquerque P.C., Nakayasu E.S., Rodrigues M.L., Frases S., Casadevall A., Zancope-Oliveira R.M., Almeida I.C., Nosanchuk D. (2008). Vesicular transport in *Histoplasma capsulatum*: An effective mechanism for trans-cell wall transfer of proteins and lipids in ascomycetes. Cell Microbiol..

[13] Alvarez F.J., Douglas L.M., Konopka J.B. (2007). Sterol-rich plasma membrane domains in Fungi. Eukaryot. Cell.

[14] Weete J.D., Abril M., Blackwell M. (2010). Phylogenetic Distribution of fungal sterols. PLoS ONE.

[15] Barreto-Bergter E., Sassaki G.L., Souza L.M. (2011). Structural analysis of fungal cerebrosides. Front. Microbiol..

[16] Guimarães L.L., Toledo M.S., Ferreira F.A., Straus A.H., Takahashi H.K. (2014). Structural diversity and biological significance of glycosphingolipids in pathogenic and opportunistic fungi. Front. Cell. Infect. Microbiol..

[17] Carrillo-Munoz A.J., Giusiano G., Ezkurra P.A., Quindos G. (2006). Antifungal agents: Mode of action in yeast cells. Rev. Esp. Quimioter..

[18] Cereghetti D.M., Carreira E.M. (2006). Amphotericin B: 50 years of chemistry and biochemistry. Synthesis.

[19] Fernández C.M., Martínez L.A., Echemendía M.Y., Martínez M.G., Perurena M.R., Illnait M.T. (2003). Sensibilidad in vitro de *Histoplasma capsulatum* var: Capsulatum frente a anfotericina B, ketoconazol, itraconazol y fluconazol. Rev. Cuba. Med. Trop..

[20] Finkelstein A., Holz R. (1973). Aqueous pores created in thin lipid membranes by the polyene antibiotics nystatin and amphotericin B. Membranes.

[21] Kaminskii D.M. (2014). Recent progress in the study of the interactions of amphotericin B with cholesterol and ergosterol in lipid environments. Eur. Biophys. J..

[22] Laniado-Laborín R., Cabrales-Vargas M.N. (2009). Amphotericin B: Side effects and toxicity. Rev. Iberoam. Micol..

[23] Hamill R.J. (2013). Amphotericin B formulations: A comparative review of efficacy and toxicity. Drugs.

[24] Stone R.H., Bicanic T., Salim R., Hope W. (2016). Liposomal Amphotericin B (AmBisome^®^): A review of the pharmacokinetics, pharmacodynamics, clinical experience and future directions. Drugs.

[25] Peláez C.A., Jiménez M.D.P., Araque P., Hung C.-Y., Castro N., Cole G.T. (2021). Lipid secretion by parasitic cells of Coccidioides contributes to disseminated disease. Front. Cell. Infect. Microbiol..

[26] Folsch J., Lees M., Sloane-Stanley G.H. (1957). A simple method for the isolation and purification of total lipids from animal tissues. J. Biol. Chem..

[27] Manocha M.S., San-Blas G., Centeno S. (1980). Lipid composition of *Paracoccidioides brasiliensis*: Possible correlation with virulence of different strains. J. Gen. Microbiol..

[28] Somasshekar D., Venkateshwaran G., Srividya C., Krishnanand S., Lokesh B.R. (2001). Efficacy of extraction methods for lipid and fatty acid composition from fungal cultures. World J. Microbiol. Biotechnol..

[29] Sepulveda J.R., Jimenez M.D.P., Peláez C.A., Araque P. (2020). Biological activity of lipids extracted from two isolates of *Fusarium oxysporum* (Environmental and Clinical) in *Galleria mellonella*. Am. J. Chem. Appl..

[30] Nelson D.L., Cuchillo Foix C.M., Lehninger A.L., Cox M.M. (2005). Lehninger: Principios de Bioquímica.

[31] Dynarowicz-Łatka P., Dhanabalan A., Oliveira O.N. (2001). Modern physicochemical research on Langmuir monolayers. Adv. Colloid Interface Sci..

[32] Sánchez-Martín M.J., Haro I., Alsina M.A., Busquets M.A., Pujol M. (2010). A Langmuir monolayer study of the interaction of E1 (145–162) hepatitis G virus peptide with phospholipid membranes. J. Phys. Chem. B.

[33] Sykora J.C., Neely W.C., Vdyanoy V. (2004). Solvent effects on amphotericin B monolayers. J. Colloid Interface Sci..

[34] Arczewska M., Gagoś M. (2011). Molecular organization of antibiotic amphotericin B in dipalmitoylphosphatidylcholine monolayer induced by K^+^ and Na^+^ ions: The Langmuir technique study. Biochim. Biophys. Acta.

[35] Maget-Dana R. (1999). The monolayer technique: A potent tool for studying the interfacial properties of antimicrobial and membrane-lytic peptides and their interactions with lipid membranes. Biochim. Biophys. Acta.

[36] Van der Meer-Janssen Y.P.M., van Galen J., Batenburg J.J., Helms J.B. (2010). Lipids in host-pathogen interactions: Pathogens exploit the complexity of the host cell lipidome. Prog. Lipid Res..

[37] Beccaccioli M., Reverberi1 M., Scala V. (2019). Fungal lipids: Biosynthesis and signalling during plant-pathogen interaction. Front. Biosci..

[38] Zarnowski R., Miyazaki M.A., Dobrzyn A., NtAmBi J.M., Woods J.P. (2007). Typing of *Histoplasma capsulatum* strains by fatty acid profile analysis. J. Med. Mycol..

[39] Murata N., Wada H. (1995). Acyl-lipid desaturases and their importance in the tolerance and acclimatization to cold of cyanobacteria. Biochem. J..

[40] Malcicka M., Visser B., Ellers J. (2018). An evolutionary perspective on linoleic acid synthesis in animals. Evol. Biol..

[41] Al-Doory Y. (1960). Free lipids and phospholipid phosphorus of *Histoplasma capsulatum* and other pathogenic fungi. J. Bacteriol..

[42] Toledo M.S., Levery S.B., Suzuki E., Straus A.H., Takahashi H.K. (2001). Characterization of cerebrosides from the thermally dimorphic mycopathogen *Histoplasma capsulatum*: Expression of 2-hydroxy fatty N-acyl (E)-∆^3^-unsaturation correlates with the yeast-mycelium phase transition. Glycobiology.

[43] Wójtowicz K., Gruszecki W.I., Walicka M., Barwicz J. (1998). Effect of amphotericin B on dipalmitoylphosphatidylcholine membranes: Calorimetry, ultrasound absorption and monolayer technique studies. Biochim. Biophys. Acta.

[44] Sandrino B., Affonso de Oliveira J.F., Nobre T.M., Appelt P., Gupta A., de Araujo M.P., Rotello V.M., Oliveira O.N. (2017). Challenges in application of Langmuir monolayer studies to determine the mechanisms of bactericide activity of ruthenium complexes. Langmuir.

