# Supplementary material for: A Langmuir-Blodgett Study of the Interaction between Amphotericin B and Lipids of Histoplasma capsulatum
Source: Membranes (Basel). 2022 Apr 29;12(5):483. doi: 10.3390/membranes12050483 (PMC9147408; doi:10.3390/membranes12050483)
Supplement: Supplementary file 1 [file membranes-12-00483-s001.zip › membranes-1695120-supplementary.pdf]

membranes

Supplementary material

A Langmuir-Blodgett Study of the Interaction between Amphotericin B and Lipids of *Histoplasma capsulatum*

Pedronel Araque-Marín [1], Andrea Naranjo Díaz [2], Luisa Fernanda Gómez Londoño [3], María del Pilar Jiménez Alzate [3], Francesco Castelli [4], Maria Grazia Sarpietro [4,*], Cristiano Giordani [5,6] and Carlos Alberto Peláez Jaramillo [2]

[1] School of Life Sciences, Universidad Escuela de Ingeniería de Antioquia (EIA), Envigado 055428, Colombia; pedronel.araque@eia.edu.co
[2] Grupo Interdisciplinario de Estudios Moleculares, Institute of Chemistry, Faculty of Exact and Natural Sciences, Universidad de Antioquia, Medellín 050010, Colombia; andrea.naranjo@udea.edu.co (A.N.D.); carlos.pelaez@udea.edu.co (C.A.P.J.)
[3] Grupo Micología Médica, Department of Microbiology and Parasitology, Faculty of Medicine, Universidad de Antioquia, Calle 70 No. 52-21, Medellín 050010, Colombia; luisa.gomezl@udea.edu.co (L.F.G.L.); delpilar.jimenez@udea.edu.co (M.d.P.J.A.)
[4] Department of Drug and Health Sciences, Università degli Studi di Catania, Viale A. Doria 6, 95125 Catania, Italy; fcastelli@unict.it
[5] Institute of Physics, Faculty of Exact and Natural Sciences, Universidad de Antioquia, Calle 70 No. 52-21, Medellín 050010, Colombia; cristiano.giordani@udea.edu.co
[6] Grupo Productos Naturales Marinos, Faculty of Pharmaceutical and Food Sciences, Universidad de Antioquia, Calle 70 No. 52-21, Medellín 050010, Colombia
* Correspondence: mg.sarpietro@unict.it

Citation: Araque-Marín, P.; Naranjo Díaz, A.; Gómez Londoño, L.F.; Jiménez Alzate, M.d.P.; Castelli, F.; Sarpietro, M.G.; Giordani, C.; Peláez Jaramillo C.A. A Langmuir-Blodgett Study of the Interaction between Amphotericin B and Lipids of *Histoplasma capsulatum*. *Membranes* **2022**, *12*, 483. https://doi.org/10.3390/membranes12050483

Academic Editor: Yukihiro Okamoto

Received: 7 April 2022
Accepted: 23 April 2022
Published: 29 April 2022

Publisher's Note: MDPI stays neutral with regard to jurisdictional claims in published maps and institutional affiliations.

Copyright: © 2022 by the authors. Licensee MDPI, Basel, Switzerland. This article is an open access article distributed under the terms and conditions of the Creative Commons Attribution (CC BY) license (https://creativecommons.org/licenses/by/4.0/).

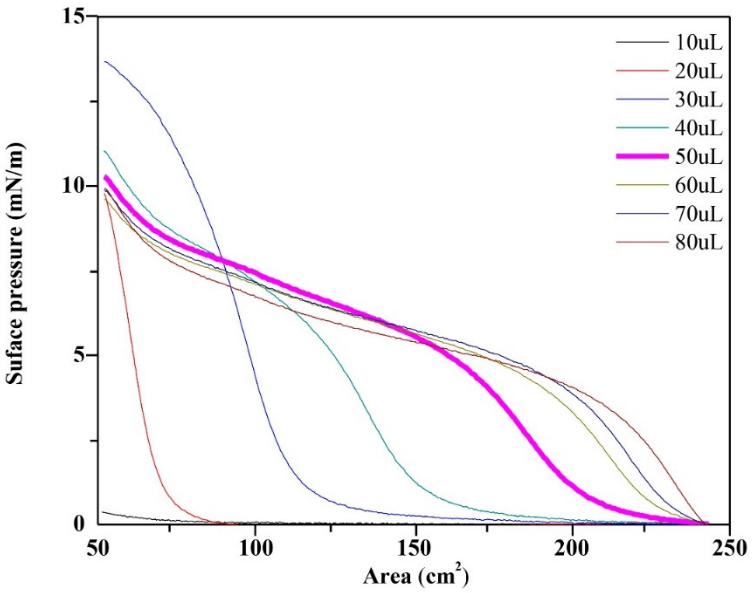

Figure S1. Surface pressure-area isotherms from the Teflon trough for the monolayers of AmB in incremental volume. Concentration is 0.447 g/L.

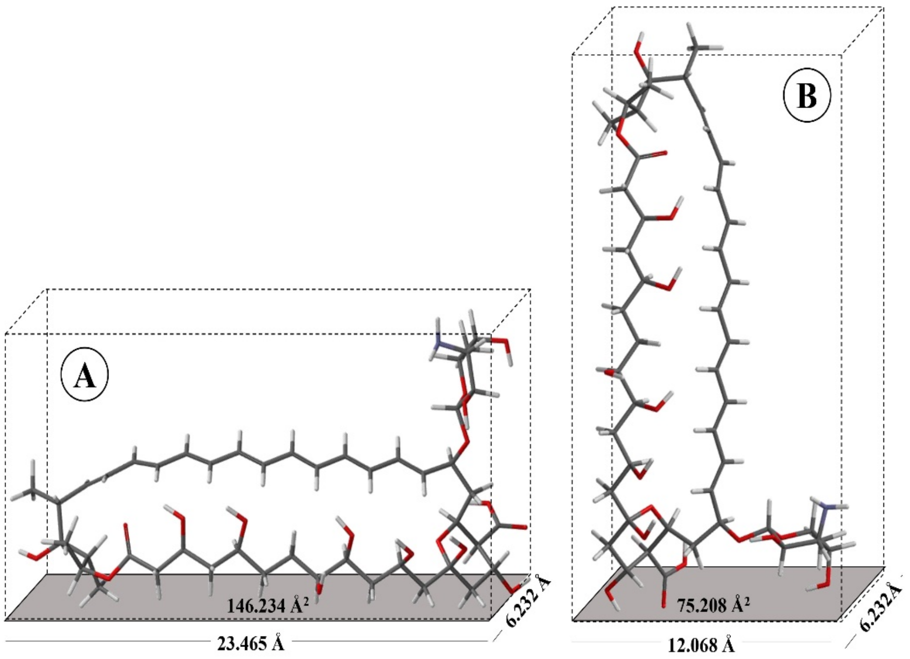

Figure S2. Reorientation model of the AmB molecule: (**A**) horizontal position (A_h); (**B**) vertical position (A_v). Energy Minimization to a value of -3271.74 kJ/mol.

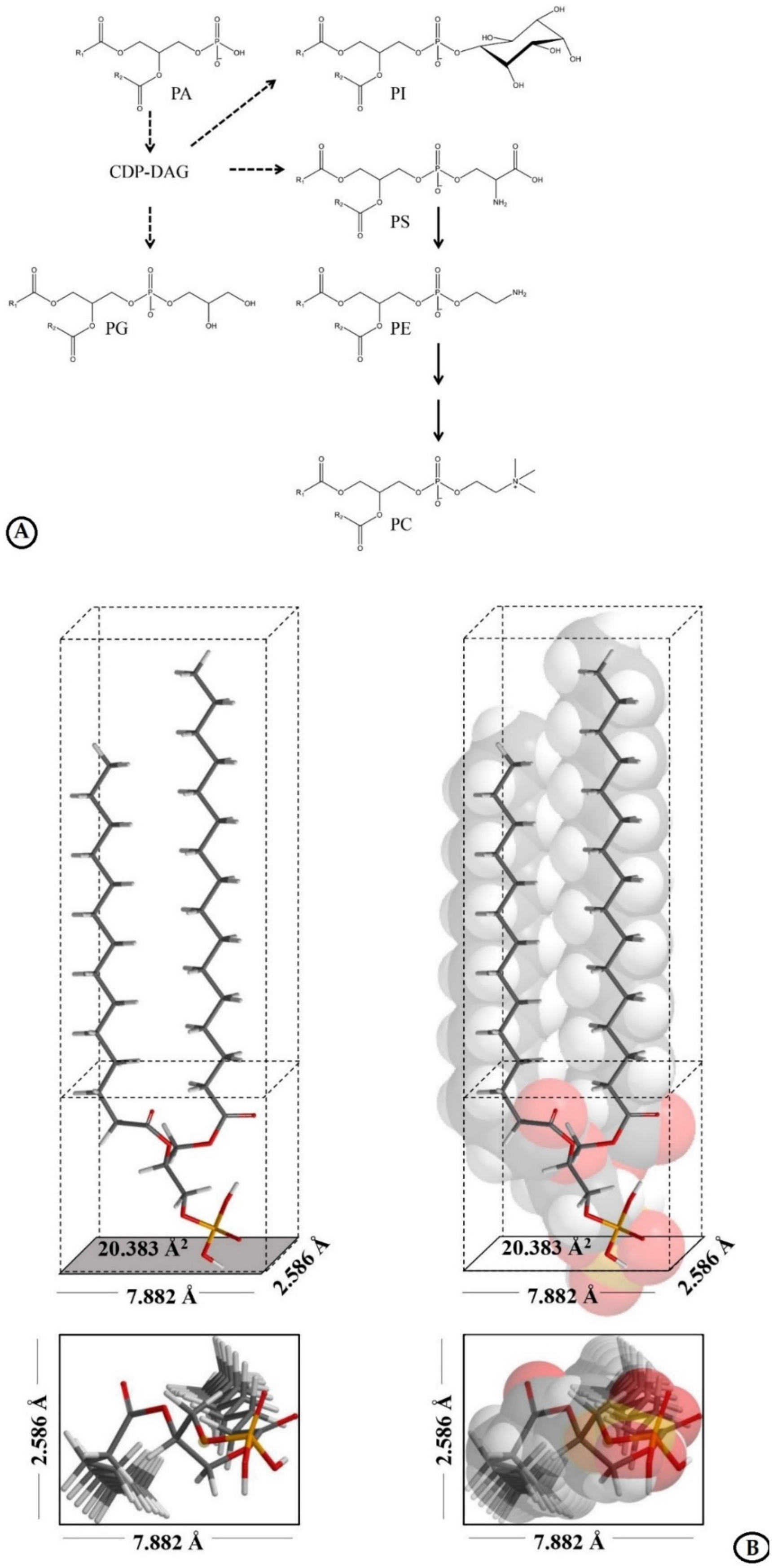

Figure S3. (**A**) Phosphatidic acid (PA) as the forerunner of the other phospholipids. (**B**) Phosphatidic acid in horizontal position (A_h) with its space-filling model and aerial views.
